# Torsion of Low-Grade Appendiceal Mucinous Neoplasm (LAMN): A Case Report

**DOI:** 10.7759/cureus.75749

**Published:** 2024-12-15

**Authors:** Hideo Kidogawa, Ryo Nonomura, Keizaburou Maruyama, Takashi Okimoto, Kohji Okamoto

**Affiliations:** 1 Department of Surgery, Kitakyushu City Yahata Hospital, Kitakyushu, JPN

**Keywords:** acute abdomen, lamn, laparoscopic surgery, low-grade appendiceal mucinous neoplasm, torsion

## Abstract

A low-grade appendiceal mucinous neoplasm (LAMN) is a rare condition, occurring in 0.08-4.1% of appendectomy cases. Although this condition is frequently asymptomatic, it may occasionally result in acute complications, including torsion and rupture. We report the case of a 33-year-old male who presented with upper abdominal pain. Contrast-enhanced computed tomography (CECT) revealed a 3 cm cystic lesion in the appendix with 360° torsion at its base. Laparoscopic surgery confirmed the torsion, and the appendix was successfully resected without mucin spillage. Histopathological examination confirmed the diagnosis of LAMN. The postoperative course was uneventful, and the patient was discharged on postoperative day four. At 2.5 years follow-up, no recurrence was observed. This case highlights the rarity of LAMN torsion and the challenges in its preoperative diagnosis. The use of advanced imaging techniques, such as CECT, is crucial in identifying the torsion preoperatively, enabling timely surgical intervention. Furthermore, the successful laparoscopic resection without mucin spillage demonstrates the importance of meticulous surgical technique in preventing complications such as Pseudomyxoma peritonei (PMP), ensuring a favorable prognosis.

## Introduction

The first description of appendiceal mucinous cystadenoma by Rokitansky in 1855 [1] marked the beginning of recognizing appendiceal lesions as distinct pathological entities, laying the groundwork for subsequent advancements in their classification and treatment. Since then, our understanding of appendiceal mucinous lesions has significantly evolved, leading to their reclassification as low-grade appendiceal mucinous neoplasm (LAMN) based on advancements in pathological and clinical research [2]. Despite its rarity, accounting for only 0.08-4.1% of appendectomy cases, the potential for misdiagnosis or under-recognition highlights the importance of understanding its clinical presentations.

LAMN is often asymptomatic and incidentally discovered. However, in rare cases, it can cause acute complications, such as torsion or rupture, leading to acute abdomen. These complications necessitate prompt diagnosis and treatment, although preoperative diagnosis can be challenging. This report highlights the importance of timely imaging and surgical intervention in the management of rare complications like torsion, providing insights into effective diagnostic and therapeutic strategies. Herein, we report a case of LAMN with torsion diagnosed preoperatively using contrast-enhanced computed tomography (CECT), followed by successful treatment.

## Case presentation

A 33-year-old male presented to our emergency center with upper abdominal pain that began the day before admission. He had been diagnosed with acute gastroenteritis at a previous clinic and received treatment, but his symptoms persisted. He denied nausea, vomiting, or diarrhea. His past medical history was unremarkable.

On examination, his vital signs were stable: blood pressure, 140/77 mmHg; heart rate, 68 bpm (regular); oxygen saturation (SpO2), 99% (room air); respiratory rate, 18 breaths/min; and body temperature, 36.8 ℃. Abdominal examination revealed decreased bowel sounds and tenderness in the upper abdomen without rebound tenderness. Laboratory findings showed an elevated white blood cell count of 13.2 × 10^3/uL (reference range: 3.3-8.6 × 10^3/uL) and a neutrophil percentage of 80.1% (reference range: 44.0-70.0%). The C-reactive protein level was 0.12 mg/dL (reference range: 0.00-0.14 mg/dL), which was normal. The rest of the blood parameters were unremarkable. These results suggested an acute inflammatory process, guiding further diagnostic imaging to identify the underlying cause.

**Figure 1:**
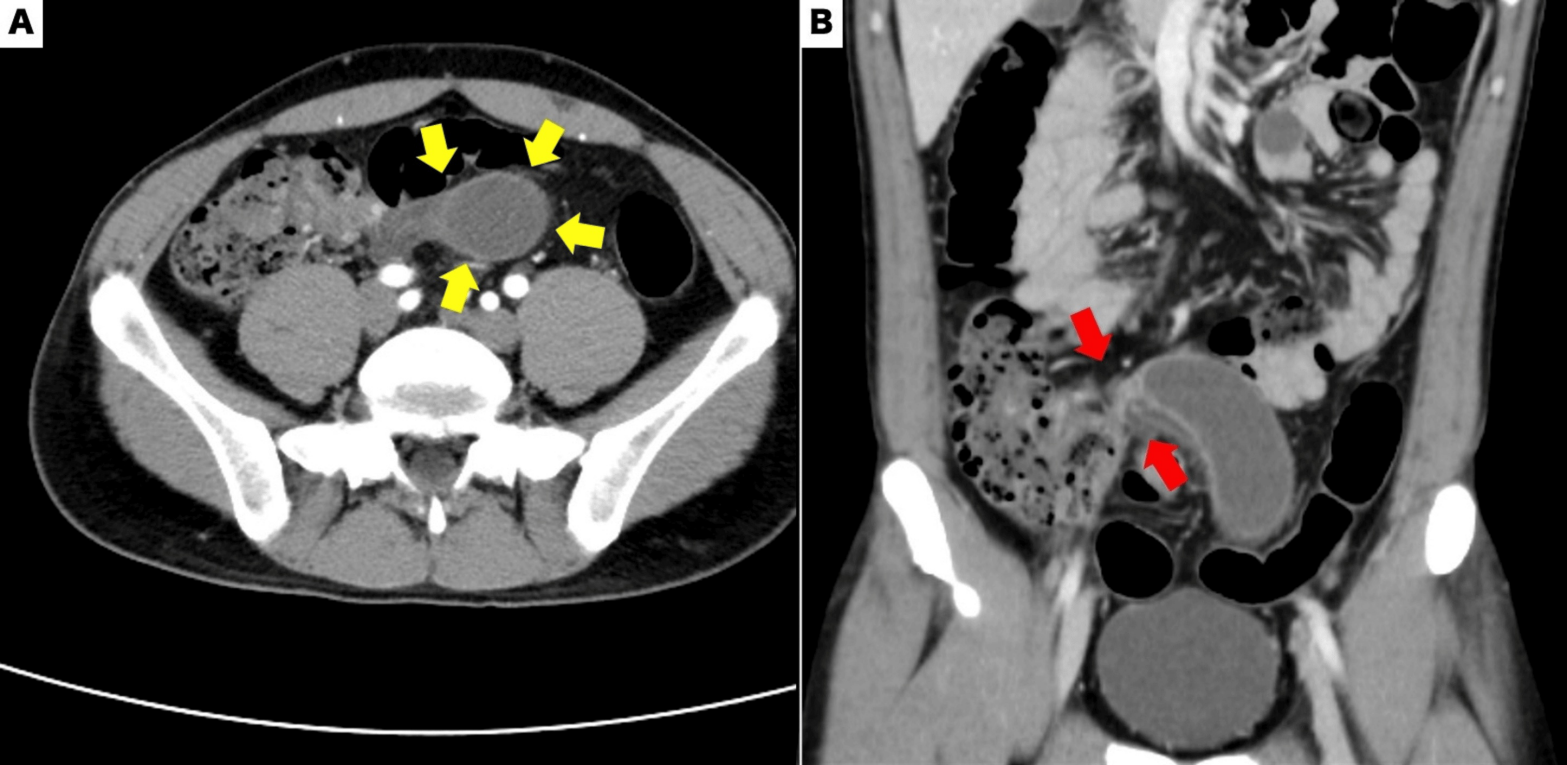
Contrast-enhanced abdominal computed tomography (CECT) A: Axial image showing a 3 cm tubular cystic lesion extending from the midline to the left lower abdomen (yellow arrow). B: Coronal image illustrating that the lesion is continuous with the medial wall of the cecum, and torsion has been identified at its base (red arrow).

CECT revealed a 3 cm tubular cystic lesion extending from the midline to the left lower abdomen (Figure 1). The lesion was continuous with the medial wall of the cecum, and torsion at its base was identified. Suspecting torsion of an appendiceal mucinous cyst, emergency laparoscopic surgery was performed.

**Figure 2:**
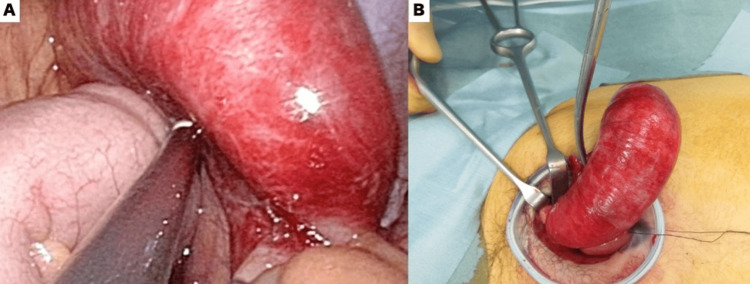
Intraoperative image A: Laparoscopic observation revealed a 360° torsion of an enlarged appendix. B: The mesoappendix was ligated and transected under direct visualization, and the appendix was double-ligated at its base and resected.

Under general anesthesia, a laparoscope was inserted through an umbilical incision, and pneumoperitoneum was established. Laparoscopic observation revealed a 360° torsion of an enlarged appendix. No ascites or purulent fluid was observed. After detorsion, the mesoappendix was grasped, and the appendix was exteriorized. The mesoappendix was ligated and transected under direct visualization, and the appendix was double-ligated at its base and resected. Careful intraoperative evaluation confirmed that the base of the appendix was intact and free of any tumor involvement or damage, allowing for an adequate resection margin to be secured. The appendix was removed intact, with no spillage of mucinous contents (Figure 2).

**Figure 3:**
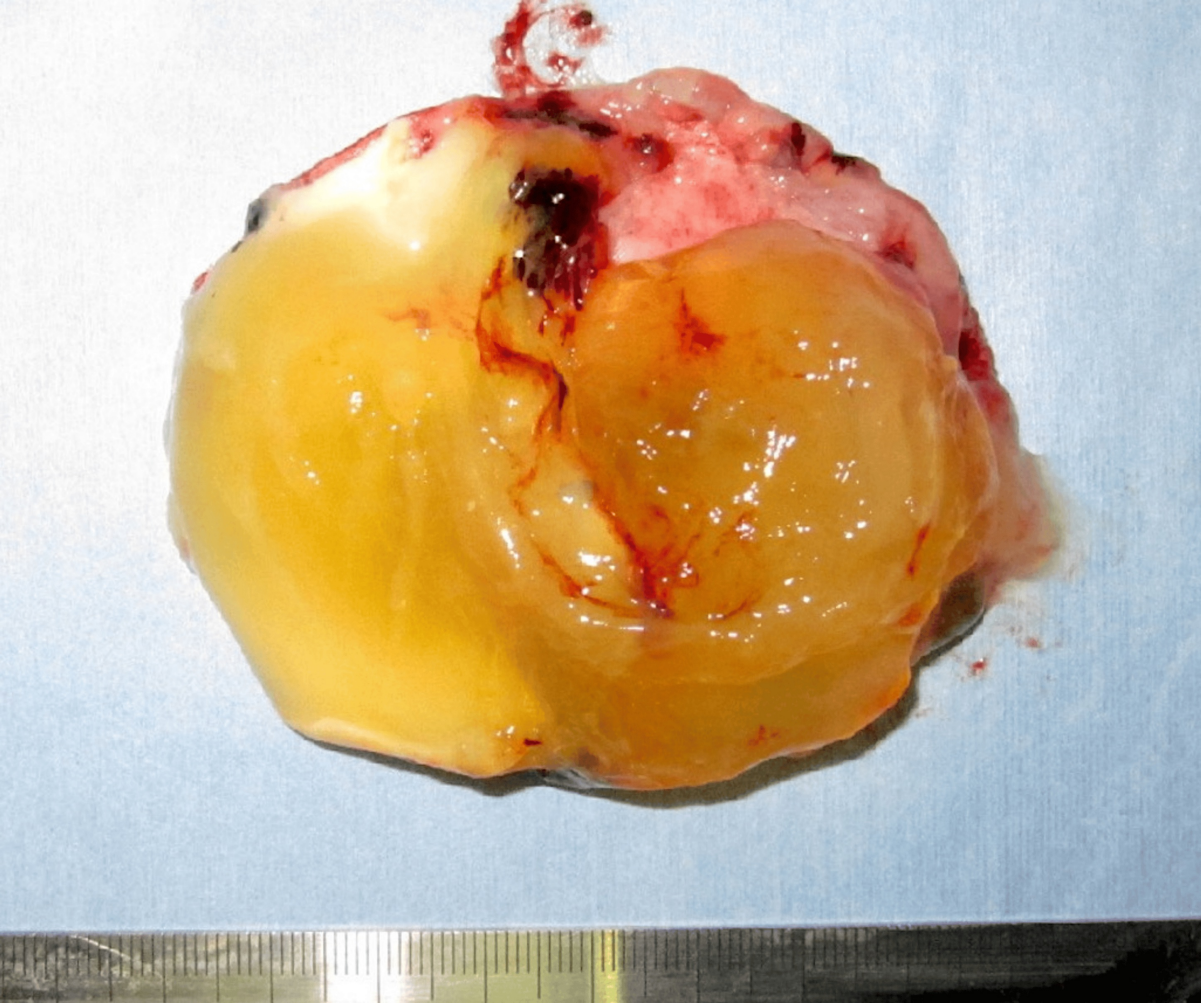
Excised specimen The excised specimen showed an appendix enlarged to 9.5 × 2.5 cm, with yellowish-white gelatinous material observed in its lumen.

The excised specimen showed an appendix enlarged to 9.5 × 2.5 cm, with yellowish-white gelatinous material observed in its lumen (Figure 3).

**Figure 4:**
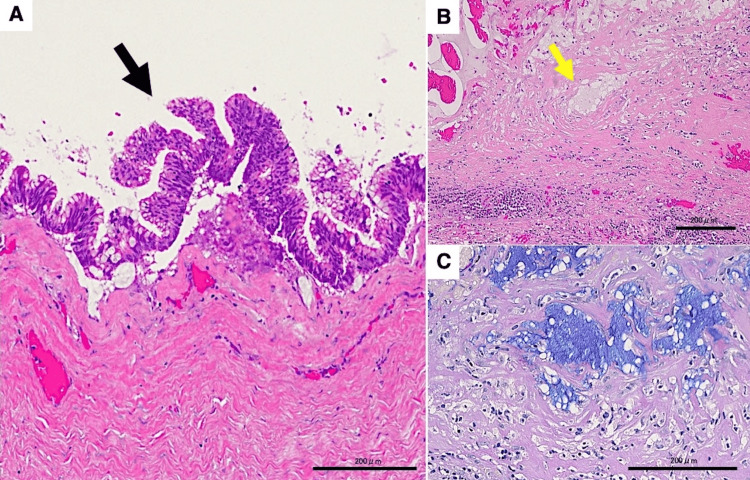
Histopathological examination (A) The luminal surface exhibited columnar epithelium with mild atypia, mucin production, and focal papillary proliferation (black arrow); (B) Pseudoinvasion of mucin pools was observed within the cyst wall, which was composed of fibrous tissue with chronic inflammatory infiltrate (yellow arrow); (C) Higher magnification of the area indicated by the arrow in panel B, stained with Alcian blue and periodic acid-Schiff, highlighting mucin pools Scale bar: 200 μm

Histopathological examination revealed a cystically enlarged appendix with extensive mucin accumulation. The luminal surface showed columnar epithelium with mild atypia, mucin production, and focal papillary proliferation. Pseudoinvasion of mucin pools was present within the cyst wall composed of fibrous tissue with chronic inflammatory infiltrate. A low-grade appendiceal mucinous neoplasm (LAMN) was diagnosed based on these findings (Figure 4).

The postoperative course was uneventful, and the patient was discharged on postoperative day four. At 2.5 years follow-up, no recurrence was observed.

## Discussion

An appendiceal mucocele is an extremely rare condition, occurring in approximately 0.08-4.1% of appendectomy cases [2]. The causes of appendiceal mucocele are diverse, ranging from benign hyperplastic lesions or obstructive cysts to borderline malignant lesions such as low-grade appendiceal mucinous neoplasm (LAMN) and even mucinous adenocarcinoma [2]. LAMN is typically asymptomatic and often detected incidentally during appendectomy or other abdominal surgeries. However, in rare cases, it can cause acute abdomen due to torsion or rupture [3,4].

The mechanism of LAMN torsion involves excessive mucin secretion due to adenomatous changes, leading to expansion of the appendiceal lumen and stretching of the mesoappendix. This process may result in mechanical stress on the appendiceal base, promoting torsion, which subsequently causes vascular impairment of the appendix [5,6]. This vascular compromise may lead to acute abdominal pain and, in severe cases, necrosis or rupture [7]. LAMN is also known to be a cause of pseudomyxoma peritonei (PMP), a condition where mucin leakage into the peritoneal cavity can lead to recurrence or disease progression [2,8]. Careful preoperative and intraoperative evaluation of the tumor’s localization and the risk of mucin leakage is crucial. In this case, CECT provided detailed information regarding the tumor’s size, location, and torsion, enabling precise preoperative planning. During surgery, meticulous intraoperative assessment confirmed that the base of the appendix was intact and free from damage or tumor involvement, ensuring the ability to secure an adequate surgical margin. These evaluations were instrumental in achieving complete tumor resection without spillage, minimizing the risk of complications such as PMP and recurrence.

Similar cases of LAMN torsion have been reported in the literature, providing valuable points of comparison. For example, Knol et al. described a case of LAMN torsion managed with laparoscopic resection, where no mucin spillage or recurrence was observed during follow-up [7]. Similarly, Ejtehadi et al. reported another case where open appendectomy was performed successfully, highlighting variations in surgical approaches [6]. These cases, along with ours, emphasize the pivotal role of preoperative imaging in accurate diagnosis and the importance of meticulous intraoperative handling to prevent complications and ensure favorable outcomes.

Imaging plays a critical role in the diagnosis of LAMN. In particular, abdominal CT can effectively depict characteristic findings of LAMN [5,6]. Typical CT findings include a cystic lesion arising from the appendix, thin walls, and homogeneous internal structures [9]. Additionally, in cases of torsion, a "whirl sign" may be observed, which is highly useful for preoperative diagnosis [5,6]. These imaging findings are not only important for diagnosing LAMN but also for identifying torsion as the cause of pain. Improved imaging techniques enable precise preoperative planning, which can significantly enhance patient outcomes.

The primary goal of LAMN treatment is complete tumor resection [7,9]. In cases with torsion, timely surgical intervention is essential to improve prognosis. During surgery, careful handling to prevent spillage of tumor contents and secure ligation of the appendiceal base are critical. Furthermore, meticulous intraoperative management to minimize mucin dissemination is believed to reduce the risk of PMP. If the tumor remains localized without ascites or dissemination, the postoperative prognosis is generally favorable [9].

In cases where tumor spillage occurs during surgery or if peritoneal metastasis or PMP is detected, additional interventions may be required. Cytoreductive surgery (CRS), including peritonectomy, is often employed to remove visible tumor deposits and improve the efficacy of subsequent therapies. Heated intraperitoneal chemotherapy (HIPEC) is typically performed after CRS to target residual tumor cells while early postoperative intraperitoneal chemotherapy (EPIC) may be used to enhance intraperitoneal therapy and further reduce the risk of recurrence. These approaches have shown promise in improving outcomes in patients with advanced appendiceal neoplasms [10].

While the prognosis of LAMN is typically good, the development of PMP is associated with a poor prognosis [2,8]. Therefore, close postoperative follow-up, including regular imaging and tumor marker assessments, is recommended to enable early detection of new lesions. While there are no established guidelines for the duration and frequency of surveillance, annual CT imaging for 5 to 10 years is generally recommended [10]. Tumor markers, such as CEA, CA19-9, and CA-125, can be useful for monitoring recurrence or progression, particularly in cases with a higher risk of PMP [10]. In cases with a high risk of recurrence or metastasis, additional treatments should be considered. The diagnosis and management of LAMN require advancements in imaging techniques and surgical approaches to improve patient outcomes. This case highlights the importance of imaging in the diagnosis and treatment of LAMN, providing valuable insights for future clinical management strategies.

## Conclusions

This case underscores the rarity of torsion as a complication of low-grade appendiceal mucinous neoplasm (LAMN) and highlights the pivotal role of preoperative imaging, particularly contrast-enhanced CT, in achieving an accurate diagnosis. The successful laparoscopic resection without mucin spillage further emphasizes the importance of meticulous surgical technique in preventing complications such as pseudomyxoma peritonei. This report provides valuable insights into the management of rare presentations of LAMN and reinforces the need for heightened awareness among clinicians to facilitate early diagnosis and intervention. Future research should focus on refining diagnostic criteria, enhancing imaging techniques, and developing standardized protocols for the management of LAMN to improve patient outcomes.
